# UBE Therapy for Dual-Segment Thoracic Disc Herniation

**DOI:** 10.1155/carm/3795148

**Published:** 2025-11-13

**Authors:** Shiwei Ren, Zhengqi Chang, Junling Pan

**Affiliations:** ^1^Department of Orthopaedics, The 960th Hospital of PLA, Jinan 250031, China; ^2^Department of Reproductive Medicine, The 960th Hospital of PLA, Jinan 250031, China

**Keywords:** case report, thoracic disc herniation, unilateral biportal endoscopy

## Abstract

This article reports a case of successful treatment of thoracic disc herniation using unilateral biportal endoscopy (UBE) technology. The patient was a young male presenting with thoracic and right rib pain for 9 months, worsening over the past 3 months. Preoperative examinations revealed protrusion of the T10/11 and T12/L1 intervertebral discs to the right, with localized calcification compressing the right nerve root. By enlarging the intervertebral foramen and utilizing UBE technology, we successfully removed the protruding calcified nucleus pulposus, significantly alleviating the patient's pain symptoms. The patient recovered well postoperatively. This case suggests that UBE technology can be a viable treatment option for patients with thoracic disc herniation.

## 1. Introduction

Thoracic disc herniation (TDH) refers to a clinical syndrome caused by various reasons (degeneration, overuse, injury, etc.), leading to the rupture of the fibrous ring of the thoracic intervertebral disc, with the nucleus pulposus tissue protruding backwards and irritating or compressing the nerve roots and spinal cord. The incidence of TDH in the general population is approximately 1/1,000,000, accounting for 0.1%–3% of all intervertebral disc herniations [1]. Symptomatic TDH accounts for less than 1% of all TDH cases, with the disease rate much lower than that of lumbar and cervical disc herniations [2]. TDH is usually located below the T7-T8 intervertebral disc, with the T11-T12 intervertebral disc being the most susceptible to degenerative changes, possibly due to the relatively weak posterior longitudinal ligament and slightly higher activity level at this level compared to other thoracic segments [3]. The majority of TDH protrusions are located in the central or posterolateral region, with less than 10% being lateral, while the occurrence of intervertebral disc calcification in TDH patients is as high as 30%–70% [4]. In clinical practice, most TDH patients undergo posterior or posterolateral thoracic decompression surgery or anterior thoracic disc excision surgery through a thoracic cavity approach. This surgical approach involves larger incisions and greater trauma, leading to increased risks of complications such as intercostal neuralgia, lung collapse, dural tears, cerebrospinal fluid leakage, and poor wound healing [4–6]. Unilateral biportal endoscopy (UBE) technology, which offers advantages such as smaller incisions, minimal trauma, clear intraoperative visibility, and faster recovery, has been widely used in spinal surgery. Recently, our department successfully treated a patient with a TDH by enlarging the intervertebral foramen and utilizing UBE technology.

## 2. Case Present

**Figure 1:**
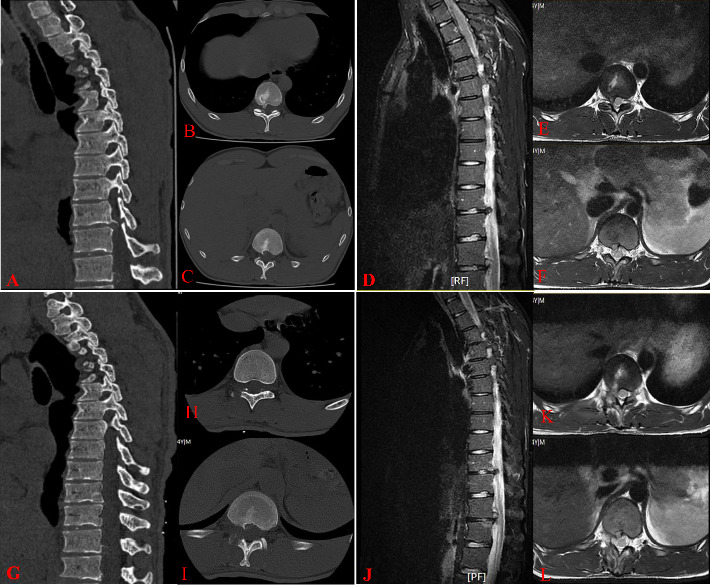
Preoperative CT and MRI scans (A–F) and postoperative follow-up CT and MRI scans (G–L).

The patient is a 24-year-old male presenting with chest and back pain accompanied by right rib pain for 9 months, worsening for the past 3 months. Positive physical examination findings include tenderness in the chest, waist, back, and right rib area, as well as decreased sensation in the skin of the right foot sole. Preoperative examinations revealed a protrusion of the T10/11 and T12/L1 intervertebral discs to the right with local calcification, compressing the right nerve root (Figures 1(A), 1(B), 1(C), 1(D), 1(E), and 1(F)). The patient's VAS score was 6, JOA score was 15, and ODI score was 33.3% before surgery. The diagnosis considered is TDH (T10/11 and T12/L1). After completing further preoperative examinations with normal blood tests, it was decided to perform UBE thoracic disc discectomy via the intervertebral foramen approach.

**Figure 2:**
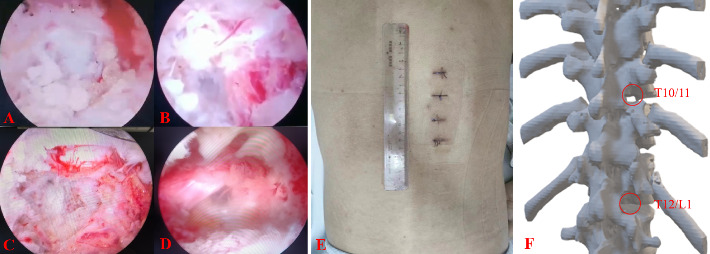
A microscopic view of the protruding nucleus pulposus and its removal (A–D), the patient's incision after surgery (E), and a three-dimensional reconstruction of the postoperative CT scan (F).

After successful general anesthesia, the patient was placed in a prone position. Under C-arm fluoroscopy guidance, the right side of the vertebral arches of T10, T11, T12, and L1 was identified. Four incisions, approximately 1 cm in size, were made at the outer and inner edges of the vertebral arches at T10/11 and T12/L1. The skin was cut open, dilating channels were installed, and a working cavity was established using a radiofrequency plasma knife under an endoscope. The protruding disc at the T10/11 level was found to be compressing the T11 nerve root, which was relieved by removing the protruding nucleus pulposus tissue (Figures 2(A) and 2(B)). A similar procedure was carried out at the T12/L1 level, where a protruding disc was removed to relieve compression on the L1 nerve root (Figures 2(C) and 2(D)). The incisions were sutured, and the surgery was completed. The operation lasted 90 min with an estimated blood loss of approximately 50 mL. The patient was instructed to wear a waistband for 24 h postoperatively and reported significant relief in back, chest, and right rib pain. The postoperative VAS score was 2, JOA score was 25, and ODI score was 13.3%, with no adverse complications reported. One week postoperatively, a follow-up chest CT and MRI were performed (Figures 1(G), 1(H), 1(I), 1(J), 1(K), and 1(L).  Figure 2(E) shows the postoperative incision, while Figure 2(F) displays a three-dimensional reconstruction of the surgical area in the thoracic spine, revealing minimal bone removal. The results from the 1-year postoperative follow-up were positive. The patient achieved a successful recovery with no evidence of common complications, specifically no infections, no instability at the surgical site, and no signs of recurrence. The overall clinical status is excellent.

## 3. Discussion

The clinical incidence of TDH is much lower than that of lumbar disc herniation and cervical disc herniation. The diagnosis of TDH is often delayed due to its slow progression and atypical clinical signs, with an average time to diagnosis of 15 months [7]. Traditional surgical methods for treating TDH include various approaches such as posterior (laminectomy), posterolateral (transpedicular and transforaminal), lateral (costotransversectomy and lateral extracavitary), lateral abdominal (thoracic, mini-thoracotomy, and thoracoscopic), and anterior (sternotomy) routes [8]. However, these traditional surgical methods have drawbacks such as large incisions, increased risk of complications such as intercostal neuralgia, lung collapse, dural tear, cerebrospinal fluid leakage, and poor wound healing. Microdiscectomy and endoscopic TDH surgery have been reported for symptomatic TDH, but they have a steep learning curve; higher risks of spinal cord injury, dural tear, and cerebrospinal fluid leakage; limited visual field; restricted decompression range; and inability to completely remove large or calcified discs [8–10].

UBE technology was first described by Italian scholar De Antoni in 1996, and the concept of UBE was first proposed by Korean scholar Heo et al. in 2017, and successfully applied in lumbar vertebral body fusion surgery [11, 12]. Compared to traditional spinal posterior fusion or laminectomy and decompression surgery, UBE enters through the muscle gap, avoiding extensive stripping of the supraspinous muscles and reducing the incidence of chronic persistent back pain after surgery. UBE can enlarge the surgical field of view and reduce the occurrence of intraoperative complications [13]. Using a water medium, UBE can maintain proper water pressure, use radiofrequency knives for hemostasis, and combine anesthesia-controlled hypotension to reduce intraoperative bleeding, resulting in a clearer field of view. By minimizing the stripping of intervertebral muscle tissue, UBE maximally preserves spinal stability, resulting in less trauma, less bleeding, and facilitating patient recovery and postoperative functional recovery [14–16]. There have been no reports of UBE surgery for thoracic disc herniation, possibly due to the risk of nerve symptoms with an inner incision or rib obstruction with an outer incision.

TDH differs from lumbar disc herniation in that effective removal of partial bone is often necessary to access the surgical site [17, 18]. Compared to uniportal endoscopic approaches, the UBE technique offers distinct advantages for managing this complex pathology. The independent viewing and working portals provide superior instrument maneuverability and a broader, more adaptable field of view, enabling highly efficient removal of obstructive bone structures and facilitating controlled foraminoplasty. In contrast, uniportal systems are limited by restricted instrument mobility and a single working channel, which hinders effective bone resection. For patients with symptomatic same-side two-level thoracic disc herniation, traditional posterior approaches carry risks such as neural injury, dural tears, extensive muscle dissection, and significant bone removal, leading to prolonged operative time and potential spinal instability. The UBE technique mitigates these risks through its muscle-sparing minimally invasive approach and exceptional visualization. Furthermore, its expanded transforaminal access allows exposure of only the lateral portion of the dura, effectively reducing the risk of neural damage.

The successful treatment of TDH in this case confirms the effectiveness and feasibility of the UBE technique for treating TDH through an enlarged intervertebral foramen approach. The procedure is thorough in decompression, minimally invasive, quick, safe, and yields good surgical outcomes. The next step is to further increase the sample size.

## Data Availability

All data analyzed during this study are included in the manuscript.
